# Nuchal Skinfold Thickness in Pediatric Brain Tumor Patients

**DOI:** 10.3389/fendo.2021.772856

**Published:** 2021-12-16

**Authors:** Junxiang Peng, Svenja Boekhoff, Maria Eveslage, Brigitte Bison, Panjarat Sowithayasakul, Carsten Friedrich, Hermann L. Müller

**Affiliations:** ^1^ Department of Pediatrics and Pediatric Hematology/Oncology, University Children’s Hospital, Klinikum Oldenburg AöR, Carl von Ossietzky University, Oldenburg, Germany; ^2^ Department of Neurosurgery, Nanfang Hospital, Southern Medical University, Guangzhou, China; ^3^ Institute of Biostatistics and Clinical Research, University of Münster, Münster, Germany; ^4^ Department of Neuroradiology, University Hospital, Würzburg, Germany; ^5^ Department of Pediatrics, Faculty of Medicine, Srinakharinwirot University, Bangkok, Thailand

**Keywords:** craniopharyngioma, obesity, pediatric brain tumor, hypothalamic involvement, skinfold thickness

## Abstract

**Background:**

Severe obesity and tumor relapse/progression have impact on long-term prognosis in pediatric brain tumor patients.

**Methods:**

In a cross-sectional study, we analyzed nuchal skinfold thickness (NST) on magnetic-resonance imaging (MRI) follow-up monitoring as a parameter for assessment of nuchal adipose tissue in 177 brain tumor patients (40 World Health Organization (WHO) grade 1–2 brain tumor; 31 grade 3–4 brain tumor; 106 craniopharyngioma), and 53 healthy controls. Furthermore, body mass index (BMI), waist-to-height ratio, caliper-measured skinfold thickness, and blood pressure were analyzed for association with NST.

**Results:**

Craniopharyngioma patients showed higher NST, BMI, waist-to-height ratio, and caliper-measured skinfold thickness when compared to other brain tumors and healthy controls. WHO grade 1–2 brain tumor patients were observed with higher BMI, waist circumference and triceps caliper-measured skinfold thickness when compared to WHO grade 3–4 brain tumor patients. NST correlated with BMI, waist-to-height ratio, and caliper-measured skinfold thickness. NST, BMI and waist-to-height ratio were associated with increased blood pressure. In craniopharyngioma patients with hypothalamic involvement/lesion or gross-total resection, rate and degree of obesity were increased.

**Conclusions:**

NST could serve as a novel useful marker for regional nuchal adipose tissue. NST is highly associated with body mass and waist-to-height ratio, and easily measurable in routine MRI monitoring of brain tumor patients.

## Introduction

Recent reports suggest that survivors of pediatric brain tumor are at increased risk of cardiovascular disease (1–4). As obesity is a well-known risk factor for the development of cardiovascular disease in the general population, this might provide a potential explanation of the added cardiometabolic risk in survivors of pediatric brain tumor (5). However, when obesity rates are analyzed based on body mass index (BMI), pediatric brain tumor patients are observed to have BMI levels similar to the general population, which is not likely to explain the observed increased risk of cardiovascular disease in pediatric brain tumor survivors (6, 7). Furthermore, as BMI is simply a measure of mass, standardized by height, it does not detail the type of the tissue present in a body. For example individuals with above-average lean (muscle) mass are frequently classified as overweight/obese, irrespective of their level of adiposity. Accordingly, more scientifically valid means of assessing body composition are available such as Dual X-ray Absorptiometry (DXA) (8, 9).

Childhood-onset craniopharyngiomas are rare malformations of embryonal origin with low-grade histological malignancy [World Health Organization (WHO) grade 1] located in the sellar/parasellar area and frequently affecting hypothalamus, pituitary gland and optic chiasm (10–12). Tumor- and/or treatment-related damage to these anatomical areas result in reduced physical and psychosocial function (13), which includes clinically severe neuroendocrine adverse effects, mainly hypothalamic obesity, with adverse influence on quality of survival after craniopharyngioma (10, 14–16). When compared with the general population, craniopharyngioma patients have a 3–19 fold higher cardiovascular mortality (17). In patients initially presenting with hypothalamic involvement of craniopharyngioma, the 20-years overall survival is reduced (13). Regular monitoring by cranial MRI to exclude recurrences are important parts of follow-up care (18).

As an important link between cardiovascular disease and obesity, regional distribution of fat in distinct compartments rather than overall obesity has been postulated. In contrast to subcutaneous adipose tissue, visceral adipose tissue is known as a fat depot, conferring metabolic risk of type 2 diabetes and atherosclerosis above and beyond standard auxiological parameters, such as waist circumference and body mass index (19–21). Upper-body subcutaneous fat, as estimated by neck circumference, may confer risk above and beyond visceral abdominal fat. Serum concentrations of free fatty acid are mainly determined by the upper-body subcutaneous fat compartment, indicating that this compartment plays an important role as specific risk factor for cardiovascular disease (22). We could previously show that nuchal skinfold thickness (NST) – as assessed in MRI of craniopharyngioma patients – serves as a predictor of metabolic risk above and beyond waist circumference and BMI in craniopharyngioma patients (23–27).

In the present study, we analyzed NST as a potential new parameter for assessment of nuchal adipose tissue and its associations with other conventional parameters for assessment of body mass and adipose tissue in long-term survivors of pediatric brain tumor.

## Materials and Methods

### Patients

In our single-center, cross-sectional study (University Children’s Hospital, Klinikum Oldenburg AöR, Germany), 177 pediatric brain tumor patients (106 craniopharyngiomas, 40 WHO grade 1–2 brain tumors; 31 WHO grade 3–4 brain tumors), recruited and longitudinally evaluated in prospective multicenter trials of the German Pediatric Brain Tumor Network (SIOP low grade glioma study –LGG, SIOP high grade glioma study – HGG; SIOP germ cell tumor study – GCT; SIOP primitive neuroectodermal study – PNET; SIOP choroid plexus tumors study – CPT Registry; KRANIOPHARYNGEOM 2000/2007) were analyzed for body height, body mass, BMI standard deviation score (SDS), and NST after a median follow-up of 2.4 years (range: 0.1–29.6 years) (28). Histological diagnoses of brain tumors and the world health organization (WHO) grading of malignancy were confirmed by neuropathological reference-assessment in all cases as part of recruitment in one of the above mentioned German brain tumor studies. The definitions of different degrees of histological malignancy (WHO grade 1-4) are based on the specific WHO criteria published for each tumor entity (29). Hypothalamic involvement and hypothalamic surgical lesions of craniopharyngioma were graded based on pre and postoperative MRI as previously described (30, 31). The control group consisted of 53 pediatric patients with normal MRI findings. In healthy controls, cranial MRIs were performed in order to exclude intracranial pathologies underlying headaches.

In 53 of 106 craniopharyngioma patients (50%), 59 of 71 brain tumor patients (83%) and 42 of 53 healthy controls (79%), associations between NST and BMI, waist-to-height ratio, caliper-measured skinfold thickness (biceps, triceps, abdominal, subscapular), and blood pressure as risk factors for cardiovascular disease could be analyzed based on complete data for all parameters. The application of anti-hypertensive medication was not assessed in all patients.

### Assessment of MRI and Anthropometric Parameters

**Figure 1:**
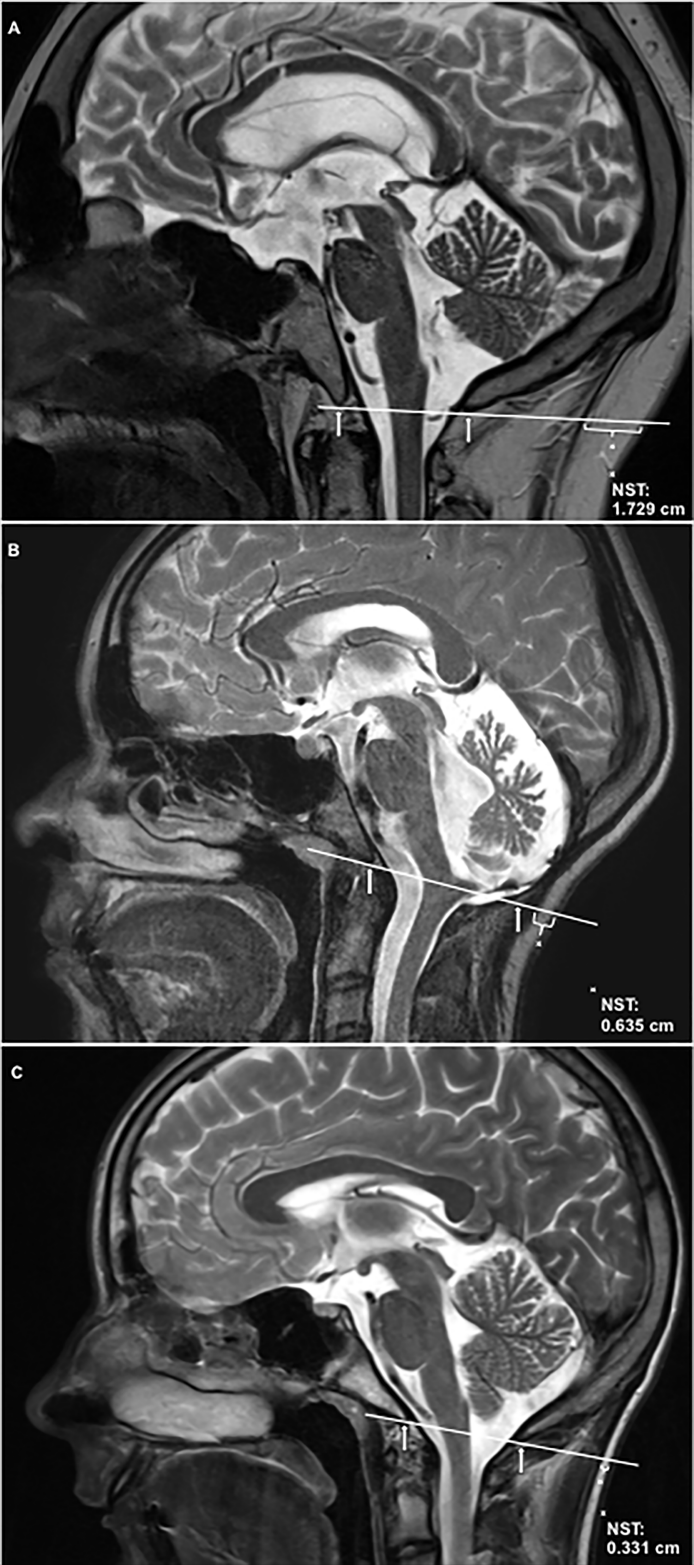
Nuchal skinfold thickness (NST) is shown on T2-weighted sagittal cranial magnetic resonance imaging (MRI) of the midline. NST was quantified according to the following standardized assessment: First a line was drawn crossing the two anatomically defined points: basion (anterior margin of the foramen magnum, indicated by arrow) and opisthion (posterior margin of the foramen magnum, indicated by arrow). The diameter of subcutaneous nuchal fat was measured over this line to the nearest 0.01 cm using OsiriX (Pixmeo SARL, Switzerland). **(A)** shows NST in a childhood-onset craniopharyngioma patient with severe obesity due to hypothalamic involvement [hypothalamic involvement grade II (13), body mass index (BMI): +4.86 SDS (32)]. **(B)** shows NST in a patient with low-grade glioma of the brain stem, BMI: +0.48 SDS. **(C)** shows NST in a healthy, normal-weight control [BMI: +0.41 SDS (32)]. SDS, standard deviation score. The inter-rater reliability of the used arithmetic mean of NST was 0.982.

NST was quantified on T1-weighted cranial MRI images of the midline performed on 1.5 Tesla MRI scanners according to a standardized procedure. First, a line was drawn crossing the two anatomically defined points: basion (anterior margin of the foramen magnum) and opisthion (posterior margin of the foramen magnum). The diameter of subcutaneous nuchal fat was measured over this line to the nearest 0.01 cm using OsiriX (Pixmeo SARL, Switzerland). Arithmetic mean of NST as measured in triplicate by three independent persons was analyzed (**Figure 1**).

Measurements of skinfold thickness at defined abdominal, subscapular, biceps and triceps areas were performed by the same person on the right side of the body using a Harpenden caliper and recorded to the nearest 0.1 cm. Waist circumferences were measured at the end of gentle expiration midway between the top of the iliac crest and the lowest rib. Waist circumference was measured over naked skin and noted to the nearest 0.1 cm. Body height was measured in triplicate using a Harpenden stadiometer and the median of three measurements was calculated as height SDS according to the Prader et al. references (33). Patients and healthy controls wearing underwear only were weighed on calibrated electronic step-scales. Body composition and the degree of obesity were evaluated by calculating the BMI SDS according to the references of Rolland-Cachera et al. (32). Systolic and diastolic blood pressure (mm Hg) were measured in seated position after resting for 15 minutes using an automatic sphygmomanometer.

### Statistical Analyses

Statistical analyses were performed with SPSS 23 for Windows (IBM Corporation, Somers, NY, USA). The intraclass correlation coefficient (ICC) was used to assess the inter-rater agreement of the NST measurements of the three raters. Groups were compared using Student’s t-test for normally distributed data, the Mann-Whitney U-test for non-normal data and Fisher’s exact tests for categorical variables. The normality assumption was verified graphically. Correlation was calculated with the Pearson correlation coefficient and corresponding 95% confidence intervals (CI). Univariate and multivariate logistic regression were applied. A stepwise selection process was used, keeping only variables with p ≤ 0.05 in the final model. Results of logistic regression are presented as odds ratio (OD) and corresponding 95%CI. All inferential statistics are intended to be exploratory (hypotheses generating), not confirmatory, and are interpreted accordingly. Therefore, no adjustment for multiple testing was applied.

### Ethics Approval

All procedures performed in our study were in accordance with the ethical standards of the institutional and/or national research committee and with the 1964 Helsinki declaration and its later amendments or comparable ethical standards. The studies KRANIOPHARYNGEOM 2000 (Clinical trial registration number: NCT00258453) and KRANIOPHARYNGEOM 2007 (Clinical trial registration number: NCT01272622) were approved by the local standing-committee on ethical practice of the Medizinische Fakultät, Julius-Maximilians-Universität Würzburg, Germany (140/99; 94/06, respectively). The current trial was approved by the local ethical committee of the Carl von Ossietzky University Oldenburg, Germany (14. January 2016; 005/2016). Written informed parental (legal guardian) and/or patient consent was obtained in all cases.

## Results

### Characteristics of Patient Cohorts and Healthy Controls

One hundred and six (60 female/46 male) of 698 childhood-onset craniopharyngioma patients (344 female/354 male) recruited in the German Childhood-onset Craniopharyngioma Registry with longitudinal follow-up in the prospective trials KRANIOPHARYNGEOM 2000 and KRANIOPHARYNGEOM 2007 were included in our study. 592 craniopharyngioma patients were excluded because one or more of the following inclusion criteria were not fulfilled: sagittal MRI of sufficient technical quality for assessment of NST, and height and weight measured within three months before or after MRI. Forty patients with brain tumor of different histology and reference-confirmed WHO grade 1 or 2 malignancy (26 low-grade glioma, 7 pituitary adenomas, 7 other histologies) and 31 patients with reference-confirmed WHO grade 3 or 4 malignancies (9 supratentorial tumors, 22 infratentorial tumors) including: 2 astrocytoma, 3 germinoma, 2 glioblastoma, 1 plexuscarcinoma, 10 medulloblastoma, 9 ependymoma, 3 diffuse intrinsic pontine glioma, 1 primitive neuroectodermal tumor (PNET) were also included in our study. Fifty-three children and adolescents (30 female/23 male) with normal cranial MRI findings, who fulfilled the above-mentioned inclusion criteria, served as healthy controls.

### Auxiological Parameters Compared Between Patient Cohorts and Healthy Controls

Childhood-onset craniopharyngioma patients were older at the time of study and presented with higher BMI SDS, NST, waist circumference, waist-to-height ratio, and caliper-measured skinfold thickness at the time of study when compared with healthy controls and patients with a brain tumor of different histology. Patients with a brain tumor of WHO grade 1 or 2 presented with higher BMI SDS, NST, waist circumference and triceps caliper-measured skinfold thickness at the time of the study when compared with WHO grade 3 or 4 brain tumor patients (**Tables 1**, **2**). With an ICC of 0.935 [95% CI (0.920; 0.948)] a good inter-rater agreement of NST measurements was observed.

**Table 1:** Characteristics of the groups of patients (106 childhood-onset craniopharyngioma, 71 childhood brain tumor patients) and 53 healthy controls and the subgroups of patients (53 craniopharyngioma, 59 brain tumor patients) and 42 healthy controls, who could be analyzed for further parameters (waist circumference, waist-to-height ratio, caliper-measured skinfold thickness) and blood pressure (systolic and diastolic).

Patients characteristics	Craniopharyngioma	Brain tumor	Healthy controls	p
**Total group (n)**	106	71	53	
**Gender (female / male)**	60 / 46	28 / 43	30 / 23	0.075
**Age at BT diagnosis (years)**	9.4 (1.3 – 20.5)	7.8 (0.1 – 17.2)		0.515
**Age at study (years)**	16.0 (2.3 – 39.0)	13.0 (1.5 – 21.0)	11.0 (3.0 – 18.0)	<0.001
**Follow-up (years)**	2.20 (0.01 – 29.59)	2.80 (0.01 –14.32)		0.728
**BMI at study (SDS)**	2.70 (-4.41 – 11.85)	0.49 (-2.87 – 14.29)	0.31 (-2.41 – 12.20)	< 0.001
**Height at study (SDS)**	-0.41 (-4.9 – 82)	0.14 (-3.74 – 3.28)	-0.10 (-3.99 – 3.67)	0.030
**Nuchal skinfold (cm)**	1.03 (0.51 – 2.74)	0.61 (0.25 – 1.59)	0.64 (0.31 – 2.17)	< 0.001
**Hypertension, n (%)**	25 (24)	18 (25)	15 (28)	0.980
**Subgroups (n)**	53	59	42	
**Gender (female / male)**	33 / 20	22 / 37	26 / 16	0.017
**Age at BT diagnosis (years)**	9.5 (1.3 – 20.5)	7.4 (0.1 – 17.2)		0.473
**Age at study (years)**	19.0 (2.3 – 35.0)	12.9 (1.5 – 18.0)	11.0 (3.0 – 17.7)	<0.001
**BMI at study (SDS)**	4.86 (-1.57 – 11.85)	0.44 (-2.14 – 14.29)	0.45 (-2.41 – 8.08)	< 0.001
**Height at Study (SDS)**	-0.10 (-3.37 – 2.64)	0.22 (-3.74 – 3.28)	0.06 (-2.93 – 3.67)	0.103
**Waist circumference (cm)**	109.0 (51.0 – 175.0)	84.0 (45.0 – 115.0)	81.5 (51.0 – 126.0)	< 0.001
**Waist-to-height ratio**	0.59 (0.38 – 0.91)	0.46 (0.37 – 0.69)	0.45 (0.35 – 0.70)	< 0.001
**Nuchal skinfold (cm)**	1.07 (0.51 – 2.74)	0.62 (0.25 – 1.59)	0.67 (0.32 – 1.98)	< 0.001
**Skinfolds (cm)**				
** abdominal**	4.20 (0.40 – 6.30)	2.10 (0.30 – 5.60)	1.50 (0.30 – 5.80)	< 0.001
** subscapular**	3.90 (0.90 – 6.50)	1.40 (0.40 – 4.80)	1.10 (0.40 – 5.20)	< 0.001
** biceps**	2.50 (0.50 – 6.00)	1.00 (0.20 – 3.80)	0.90 (0.30 – 2.80)	< 0.001
** triceps**	3.10 (0.90 – 6.00)	1.60 (0.50 – 4.80)	1.45 (0.60 – 4.30)	< 0.001
**RR systolic (mm HG)**	128 (99 – 176)	118 (80 – 171)	118 (87 – 167)	0.014
**RR diastolic (mm HG)**	82 (54 – 143)	72 (47 – 110)	74 (50 – 104)	0.001
**Hypertension, n (%)**	20 (38)	16 (27)	13 (31)	0.487

BT-Normal Controls: BMI SDS p=0.750 (total group); BT-Normal Controls: BMI SDS p=0.962 (Subgroups).

Shown are median and (ranges). BMI, body mass index, BT, brain tumor; RR, blood pressure; mm HG, millimeter mercury; SDS, standard deviation score; at study means “at the time of cranial MRI”.

**Table 2:** Patients’ characteristics in childhood brain tumor (BT) patients with regard to histological grade of malignancy according to WHO grading system (grade 1 to 4).

Patients characteristics	Grade 1-2 BT	Grade 3-4 BT	p
**Total group (n)**	40	31	
**Gender (female / male)**	18 / 22	10 / 21	0.332
**Age at BT diagnosis (years)**	8.0 (0.5 – 17.2)	7.7 (0.1 – 17.0)	0.692
**Age at study (years)**	13.1 (2.0 – 21.0)	13.0 (1.5 – 19.0)	0.719
**BMI at study (SDS)**	+1.04 (-2.87 – 14.29)	-0.01 (-2.14 – 4.62)	0.048
**Height at study (SDS)**	0.60 (-3.74 – 3.28)	-0.30 (-2.98 – 2.11)	0.173
**Nuchal skinfold (cm)**	0.66 (0.34 – 1.59)	0.58 (0.25 – 1.10)	0.043
**Hypertension, n (%)**	13 (33)	5 (16)	0.105
**Subgroups (n)**	32	27	
**Gender (female / male)**	14 / 18	8 / 19	0.293
**Age at BT diagnosis (years)**	7.9 (0.5 – 17.2)	7.2 (0.1 – 16.5)	0.585
**Age at study (years)**	12.7 (4.8 – 18.0)	13.0 (1.5 – 18.0)	0.796
**BMI at study (SDS)**	1.21 (-1.13 – 14.29)	-0.11 (-2.14 – 4.62)	0.047
**Height at study (SDS)**	0.72 (-3.74 – 3.28)	-0.30 (-2.98 – 2.11)	0.079
**Waist circumference (cm)**	87.0 (55.0 – 115.0)	75.5 (45.0 – 107.0)	0.048
**Waist-to-height ratio**	0.47 (0.37 – 0.69)	0.44 (0.37 – 0.60)	0.639
**Nuchal skinfold (cm)**	0.65 (0.34 – 1.59)	0.52 (0.25 – 1.10)	0.169
**Skinfolds-caliper (cm)**			
** abdominal**	1.60 (0.30 – 5.50)	1.30 (0.60 – 5.60)	0.090
** subscapular**	1.60 (0.40 – 4.80)	1.30 (0.50 – 4.00)	0.242
** biceps**	1.25 (0.20 – 3.40)	0.90 (0.40 – 3.80)	0.220
** triceps**	2.10 (0.60 – 3.80)	1.30 (0.50 – 4.80)	0.016
**RR systolic (mm HG)**	121 (96 – 171)	114 (80 – 141)	0.065
**RR diastolic (mm HG)**	74 (47 – 97)	70 (52 – 110)	0.135
**Hypertension, n (%)**	11 (34)	5 (18)	0.242

Shown are median and (ranges). RR, blood pressure; mm HG, millimeter mercury; BT, brain tumor; SDS, standard deviation score; WHO, World Health Organization; at study means “at the time of cranial MRI”.

**Figure 2:**
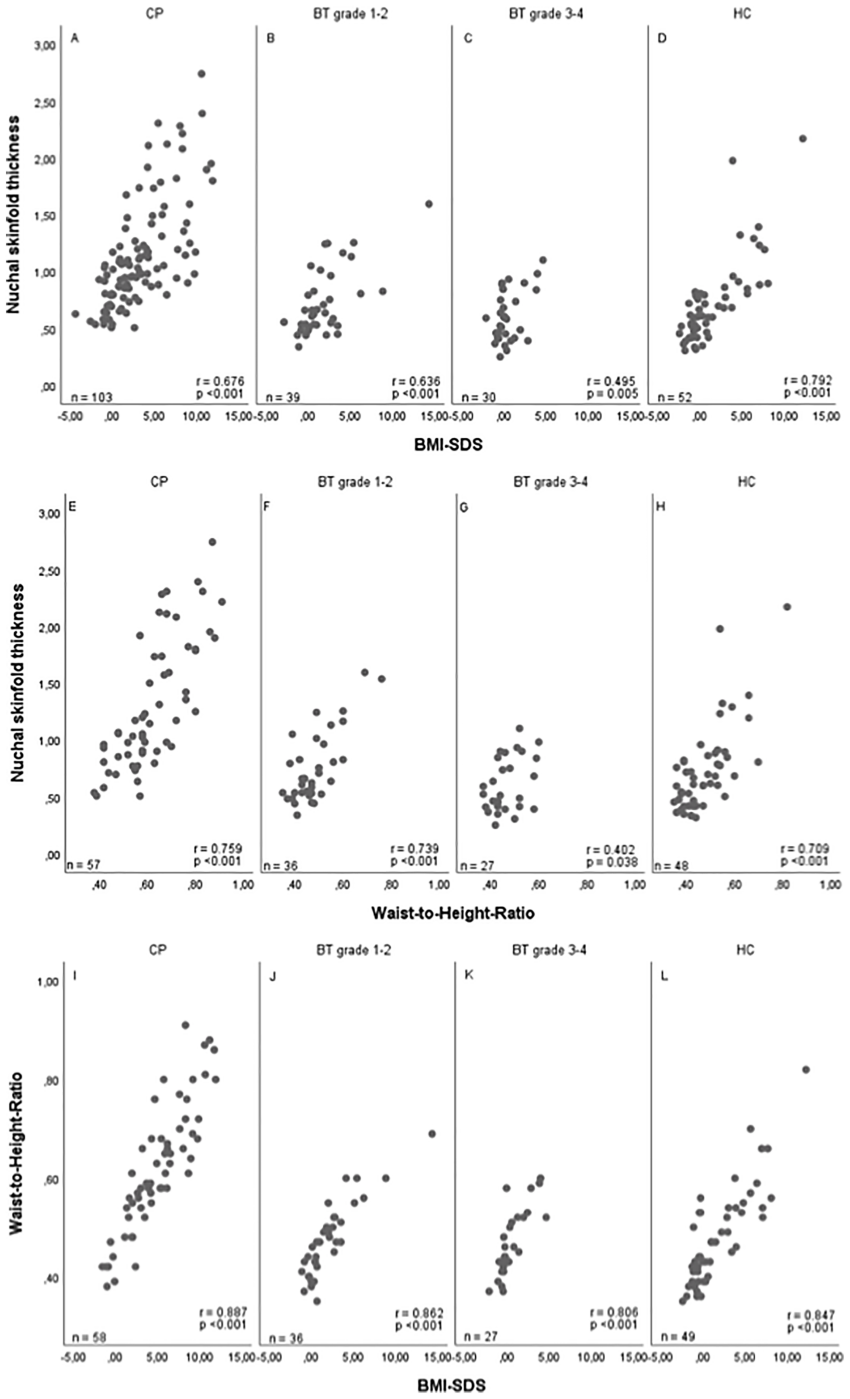
Correlations between nuchal skinfold thickness (NST) as measured (cm) on cranial magnetic resonance (MRI) imaging and body mass index (BMI) SDS (32) **(A–D)**, between NST and waist-to-height ratio **(E–H)**, and between BMI SDS and waist-to-height ratio **(I–L)** in patients with childhood-onset craniopharyngioma (CP) recruited in HIT Endo and KRANIOPHARYNGEOM 2000/2007 **(A, E, I)**, WHO grade 1 or 2 brain tumor (BT) patients **(B, F, J)**, WHO grade 3 or 4 BT patients **(C, G, K)**, and healthy controls (HC) **(D, H, L)**. r = Pearson correlation coefficient; SDS, standard deviation score.

High positive correlations were observed between NST and BMI SDS and NST and waist-to-height ratio: NST *vs*. waist-to-height ratio: r=0.804, 95%CI (0.744–0.852); BMI SDS *vs.* waist-to-height ratio: r=0.878, 95%CI (0.839–0.909); NST *vs.* BMI SDS: r=0.716, 95%CI (0.645–0.774). The results in all patient subgroups and healthy controls are shown in **Figure 2**. The different ranges of BMI SDS, waist-to-height ratio and NST values lead to differences in correlation coefficients between the subgroups that do not necessarily reflect different underlying associations.

### Caliper-Measured Skinfold Thickness

**Figure 3:**
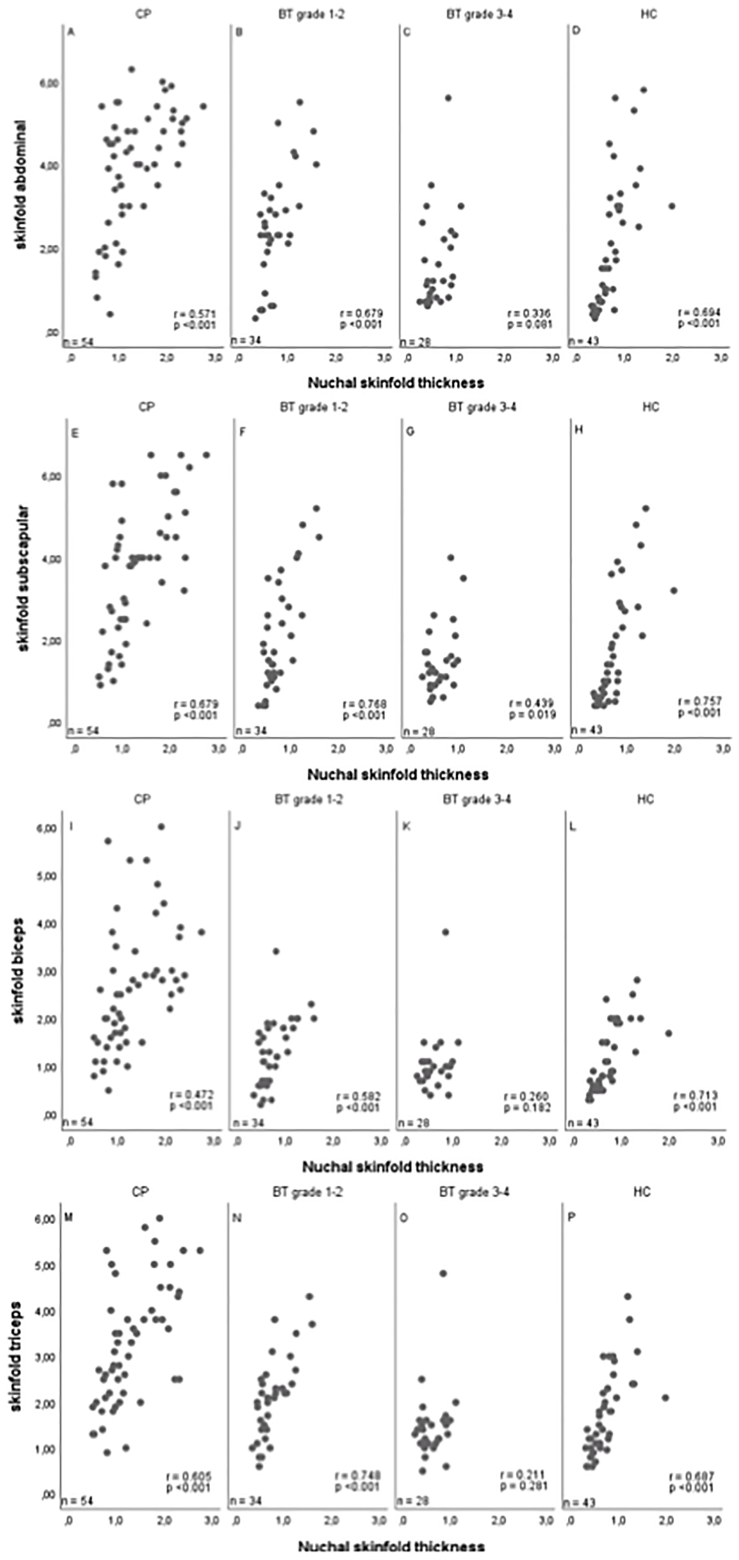
Correlations between nuchal skinfold thickness (NST) as measured (cm) on cranial magnetic resonance imaging (MRI) and caliper-measured skinfold thickness for abdominal **(A–D)**, subscapular **(E–H)**, biceps **(I–L)** and triceps **(M–P)** skinfold thickness in patients with childhood-onset craniopharyngioma (CP) **(A, E, I, M)**, WHO grade 1 or 2 brain tumor (BT) patients **(B, F, J, N)**, WHO grade 3 or 4 BT patients **(C, G, K, O)**, and healthy controls (HC) **(D, H, L, P)**. r = Pearson correlation coefficient.

In the subgroups of 53 craniopharyngioma patients, 32 patients with WHO grade 1 or 2 brain tumor, 27 patients with WHO grade 3 or 4 brain tumor, and 42 healthy controls, associations between NST, waist-to-height ratio, caliper-measured skinfold thickness (abdominal, subscapular, biceps, triceps), BMI, and blood pressure could be analyzed (**Table 1**). Also in these subgroups, NST correlated with BMI SDS and waist-to-height ratio: BMI SDS *vs*. NST: r=0.743 95%CI (0.663–0.807); BMI SDS *vs*. waist-to-height ratio: r=0.885, 95%CI (0.846–0.915); waist-to-height ratio *vs*. NST: r=0.793, 95%CI (0.726–0.845). Comparing NST with caliper-measured skinfold thickness, high correlations between NST and all assessed caliper-measured skinfold thicknesses were observed: NST *vs*. abdominal caliper-measured skinfold thickness: r=0.705, 95%CI (0.617–0.776); NST *vs*. biceps caliper-measured skinfold thickness: r=0.677, 95%CI (0.583–0.753); NST *vs.* triceps caliper-measured skinfold thickness: r=0.733, 95%CI (0.653–0.798); NST *vs.* subscapular caliper-measured skinfold thickness: r=0.783, 95%CI (0.715–0.837) (**Figure 3**).

The analyses depicted in **Figure 3** were calculated for all pairwise non-missing observations, therefore the sample sizes may differ between **Tables 1**, **2**.

**Figure 4:**
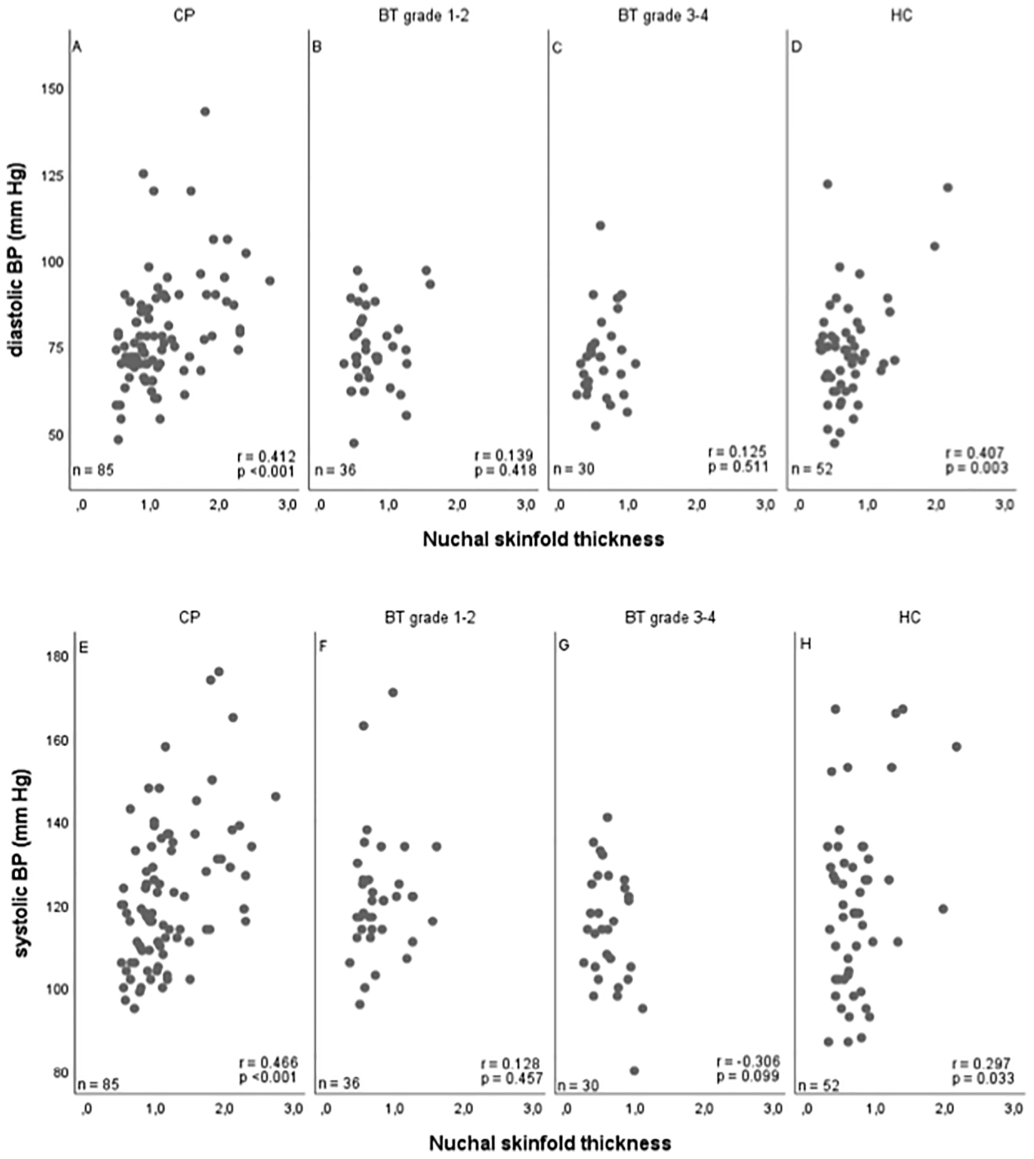
Correlations between nuchal skinfold thickness (NST) as measured (cm) on cranial magnetic resonance imaging (MRI) and diastolic **(A–D)** and systolic **(E–H)** blood pressure (BP) (mm Hg) in patients with childhood-onset craniopharyngioma (CP) **(A, E)**, WHO grade 1 or 2 brain tumor (BT) patients **(B, F)**, WHO grade 3 or 4 BT patients **(C, G)**, and 43 healthy controls (HC) **(D, H)**. r = Pearson correlation coefficient; mm HG, millimeter mercury.

Systolic blood pressure correlated with NST (r=0.327, 95%CI 0.198–0.444), BMI SDS (r=0.387; 95%CI 0.263–0.497), and waist-to-height ratio (r=0.266; 95%CI 0.119-0.400). Similar results were observed for diastolic blood pressure, showing that also diastolic blood pressure correlated with NST (r=0.400, 95%CI 0.278–0.510), BMI SDS (r=0.417, 95%CI 0.297–0.524), and waist-to-height ratio (r=0.352; 95%CI 0.212–0.478) (**Figure 4**).

### Hypothalamic Involvement

Seenty-four of 85 craniopharyngioma patients (87%) with available data presented with hypothalamic involvement of craniopharyngioma at the time of diagnosis, which was associated with obesity. Patients with hypothalamic involvement presented with higher BMI SDS (median BMI: +2.15 SDS, range: -4.41 to +11.85 SDS, p=0.001), higher NST (median NST: 1.02 cm, range: 0.51–2.74 cm, p=0.017), and higher waist-to-height ratio (median waist-to-height ratio: 0.58, range: 0.38–0.87, p=0.004), when compared to craniopharyngioma patients without hypothalamic involvement (median BMI: -0.31 SDS, range: -1.57 to +3.30 SDS; median NST: 0.77 cm, range: 0.51–1.13 cm; median waist-to-height ratio: 0.42, range: 0.39–0.44). In our subgroups of brain tumor patients with histological diagnoses different from craniopharyngioma, no cases with hypothalamic involvement were observed.

### Degree of Surgical Resection

Twenty-one of 96 craniopharyngioma patients (22%) were treated by gross-total resection achieving reference-confirmed complete tumor removal. After gross-total resection, patients presented with higher BMI SDS (median BMI: +4.86 SDS, range: -1.57 to +10.54 SDS, p=0.008), NST (median: 1.12 cm, range: 0.51–2.74 cm, p=0.305) and waist-to-height ratio (median: 0.65, range: 0.39–0.91, p=0.385) at time of study when compared with patients after incomplete resection (median BMI: +1.79 SDS, range: -4.41 to +11.85 SDS; median NST: 1.00 cm, range: 0.54–2.39 cm; median waist-to-height ratio: 0.59, range: 0.38–0.86).

### Surgical Hypothalamic Lesions

Forty-eight of 87 craniopharyngioma patients (55%) with available data for surgical hypothalamic lesions presented with post-surgical hypothalamic lesions. Patients with hypothalamic lesions presented with higher BMI SDS (median: +3.16 SDS, range: -4.41 to +11.85 SDS, p<0.001), higher NST (median: 1.11 cm, range: 0.54–2.74 cm, p<0.001), and waist-to-height ratio (median: 0.64, range: 0.42–0.87, p=0.001) when compared to the subgroup of craniopharyngioma patients without hypothalamic lesions (BMI SDS median: +0.87 SDS, range: -2.63 to +11.68 SDS; NST median: 0.91 cm, range: 0.51–1.95 cm; waist-to-height ratio median: 0.55, range: 0.38–0.86).

### Multivariate Analysis of Risk Factors for Hypertension

In multivariate analyses including 53 craniopharyngioma patients, 59 brain tumor patients and 42 healthy controls, we analyzed which of the anthropometric parameters NST, BMI SDS, waist-to-height ratio, caliper-measured skinfold thickness had potential impact on blood pressure as a risk factor for cardiovascular disease. In all patients and healthy controls, systolic and diastolic blood pressure values were adjusted for age, gender and height and classified as normotensive or hypertensive blood pressure according to a study on waist-to-height ratio and elevated blood pressure (34). However, antihypertensive medication was not assessed sufficiently in all patients, which might influence our results by potential underestimation of hypertensive cases. When analyzing the total group of 154 participating patients/healthy controls, several parameters such as waist-to-height ratio, caliper-measured skinfold thickness, NST, waist circumference and BMI SDS could be identified as potential risk factors for hypertension in univariable analysis (data not shown). When entering all anthropometric parameters in a multivariable logistic regression analysis and performing stepwise selection, only BMI SDS was selected for the final model resulting in an odds ratio of 1.25, 95%CI (1.14–1.37). Of course, this analysis was strongly limited by the small cohort size and also the high correlation of the anthropometric measures.

## Discussion

Metabolic syndrome consisting of insulin resistance and a minimum of two other risk factors from increased BMI, elevated blood pressure, hypertriglyceridemia, low serum HDL-cholesterol, and microalbuminuria, can result in cardiovascular disease. Obese children have an increased risk of metabolic syndrome, due to their large compartment of fat tissue when compared with children with normal body mass (35–38). There are several distinct fat tissue compartments (subcutaneous adipose tissue, visceral adipose tissue, white adipose tissue present intramuscularly, and brown adipose tissue commonly found in the supraclavicular and subscapular regions) are known with different metabolic characteristics (39). Our study was focused on nuchal adipose tissue, which has been described as a risk factor for metabolic syndrome and has a strong association with the risk of cardiovascular disease (23, 24).

MRI is the gold standard to quantify compartments of different adipose tissue. However, MRI is an expensive method to be performed for this purpose alone. The value and feasibility of auxiological parameters for risk prediction of metabolic syndrome and cardiovascular disease have been analyzed by several studies. In a study of adults, where the visceral adipose tissue on MRI was used as reference, waist circumference correlated better with visceral adipose tissue than BMI, which seemed to be more associated with total adipose tissue (40, 41). A study of Koren et al. (42) showed that in adolescents the sagittal abdominal diameter, which represents abdominal thickness at waist level in supine position, was a better predictor of visceral adipose tissue than waist-to-hip ratio, waist circumference, and BMI. Although in adults the waist-to-hip ratio is associated with cardiovascular disease and type 2 diabetes, this association is less clear in the pediatric age group, most likely due to changes in distribution of body fat during growth (43, 44). Several studies could show that the ratio of waist circumference to height (waist-to-height ratio) is superior to BMI and waist circumference to predict risks of cardiovascular disease (45–48). In a study of adolescents, waist-to-height ratio was associated with BMI SDS and both waist-to-height ratio and BMI had predictive value for arterial hypertension (34). An advantage of waist-to-height ratio measurement is that waist-to-height ratio does not need adjustment for age: a cut-off value of 0.5 can be used in every age group. This makes waist-to-height ratio usable for comparison in pediatric patients of different age (34, 49).

Preis et al. (23) and Da Silva et al. (50) reported on neck circumference as a new parameter for prediction of cardiovascular disease risk. Preis et al. (23) could show that neck circumference was correlated with both BMI and visceral adipose tissue. After adjustment for visceral adipose tissue, the authors observed that neck circumference was positively correlated with blood pressure and risk factors for cardiovascular disease (23).

Long-term prognosis after pediatric brain tumor disease is frequently impaired by cardiovascular disease (1). Craniopharyngioma patients have a 3–19 fold higher cardiovascular mortality when compared with the general population (17) due to hypothalamic obesity caused by tumor and/or treatment related hypothalamic lesions (10, 13–16). Incidence of cardiovascular disease is also increased in patients with pediatric brain tumor of other histology. However, when obesity rates of patients with a brain tumor different from craniopharyngioma are measured by using BMI, pediatric brain tumor patients were observed to present with BMI levels that were either close to or slightly higher than BMI in the general population (6, 7). In accordance with previous reports (6, 7), we also observed no BMI differences between brain tumor patients and healthy controls in our study. However, patients with WHO grade 1–2 brain tumor presented with higher BMI when compared with WHO grade 3-4 brain tumor patients, indicating that low histological grade of malignancy and different treatment might have impact on body composition.

In our previous study (27), analyzing NST on cranial MRIs of craniopharyngioma patients performed during follow-up monitoring, we reported on NST as a new parameter for body composition assessment. We observed that NST was associated with other parameters of body composition such as BMI, caliper-measured skinfold thickness, and waist-to-height ratio. Furthermore, NST had predictive value for cardiovascular disease risk in craniopharyngioma patients and healthy controls.

In this study, we analyzed NST and its associations with the above mentioned anthropometric parameters not only in larger cohorts of craniopharyngioma and healthy controls, but also in pediatric patients with a brain tumor of different histological diagnosis and grades of malignancy. In cross-sectional analyses, NST correlated with the anthropometric parameters BMI and caliper-measured skinfold thickness and was associated with waist-to-height ratio as a known parameter of visceral adipose tissue in all patient groups and in healthy controls. Craniopharyngioma patients with hypothalamic involvement presented with the highest NST. Furthermore, our observations support previous reports (13, 14) that hypothalamic involvement and hypothalamic lesions are associated with severe obesity in long-term survivors of childhood-onset craniopharyngioma.

The results of our study add to the literature on sequelae after brain tumor disease by reporting on the novel observation that NST is a reliable parameter of nuchal adipose tissue correlating with body mass (BMI SDS, waist-to-height ratio) not only in craniopharyngioma patients but also in patients with a brain tumor of different histology and grade of malignancy. Assessment of NST is easy to perform based on a standardized procedure with high reliability. Limitations of our pilot study are differences with regard to older age of craniopharyngioma patients at the time of study/MRI and the small cohort size of patients with a pediatric brain tumor other than craniopharyngioma, which warrants further analyses of larger age-matched cohorts. A further limitation relates to the fact, that due to the high rate of eating disorders in craniopharyngioma patients fasting blood samples for assessment of other risk factors for cardiovascular disease such as glycemic control or lipid status were not available. Furthermore, assessment of total body adiposity could not be achieved, as no sites on the lower body compartment were assessed. Given that impedance analyses are known to be relatively invalid (from variation due to hydration status and food consumption amongst other factors), standardized procedures for medical imaging such as MRI and dual-energy X-ray absorptiometry (DXA) are preferred for VAT measurement. HOMA-IR and other measures of visceral adiposity (i.e. impedance analyses, abdominal MRI, DXA) could not be evaluated in this study but will be part of future prospective studies in context of the German Craniopharyngioma Registry.

## Conclusions

MRI-based assessment of NST is not recommended as a routine clinical procedure for assessment of nuchal adipose tissue in obese patients. However, as cranial MRI plays an important role in routine follow-up monitoring for early detection of recurrent brain tumor disease, measurement of NST could serve as a reliable, standardized and easily determinable parameter for nuchal adipose tissue during follow-up after brain tumor. Based on the high association of NST with conventional parameters of body mass, NST might be a suitable tool for identifying brain tumor patients at risk of post-treatment obesity. Especially with regard to the difficulties in collecting reliable anthropometric data in the multicenter setting of our national registry, NST on MRI provides a promising alternative. Furthermore, we could demonstrate that pediatric patients with a brain tumor of low histological malignancy (WHO grade 1 or 2) and craniopharyngioma patients with hypothalamic involvement are at special risk for obesity.

## Data Availability Statement

The datasets generated or analyzed during this study are available from the corresponding author on reasonable request.

## Ethics Statement

The studies involving human participants were reviewed and approved by Ethical committee of the medical faculty of the Carl von Ossietzky University, European Medical School (EMS), Oldenburg, Germany. The patients/participants provided their written informed consent to participate in this study.

## Author Contributions

JP researched the data and wrote the manuscript. SB collected the data and prepared statistical analyses, contributed to the analytical plan and discussion and reviewed/edited the manuscript. ME performed all statistical analyses, contributed to the analytical plan and discussion and reviewed/edited the manuscript. BB did neuroradiological assessment of all imaging. BB was the neuroradiologist, who performs reference-assessment of imaging in all patients recruited in KRANIOPHARYNGEOM 2000/2007. She prepared the imaging data and their presentation and reviewed/edited the manuscript. PS, and CF contributed to the analytical plan and discussion and reviewed/edited the manuscript. HM initiated and conducted the multicenter trials KRANIOPHARYNGEOM 2000 and KRANIOPHARYNGEOM 2007, contributed to the analytical plan and discussion and reviewed/edited the manuscript. Assessments of NST were performed in triplicate (by JP, SB, and HM) for each patient.

## Funding

This study was funded by grants (HM, DKS2014.13, BB, DKS2018.02) of the German Childhood Cancer Foundation, Bonn, Germany.

## Conflict of Interest

HM has received reimbursement of participation fees for scientific meetings and continuing medical education events from the following companies: Ferring, Lilly, Pfizer, Sandoz/Hexal, Novo Nordisk, IPSEN, and Merck Serono. He has received reimbursement of travel expenses from IPSEN and lecture honoraria from Pfizer.

The remaining authors declare that the research was conducted in the absence of any commercial or financial relationships that could be construed as a potential conflict of interest.

## Publisher’s Note

All claims expressed in this article are solely those of the authors and do not necessarily represent those of their affiliated organizations, or those of the publisher, the editors and the reviewers. Any product that may be evaluated in this article, or claim that may be made by its manufacturer, is not guaranteed or endorsed by the publisher.
